# Type I Interferons Are Involved in the Intracellular Growth Control of *Mycobacterium abscessus* by Mediating NOD2-Induced Production of Nitric Oxide in Macrophages

**DOI:** 10.3389/fimmu.2021.738070

**Published:** 2021-10-28

**Authors:** Jae-Hun Ahn, Ji-Yeon Park, Dong-Yeon Kim, Tae-Sung Lee, Do-Hyeon Jung, Yeong-Jun Kim, Yeon-Ji Lee, Yun-Ji Lee, In-Su Seo, Eun-Jung Song, Ah-Ra Jang, Soo-Jin Yang, Sung Jae Shin, Jong-Hwan Park

**Affiliations:** ^1^ Laboratory Animal Medicine, College of Veterinary Medicine and BK21 FOUR Program, Chonnam National University, Gwangju, South Korea; ^2^ Department of Veterinary Microbiology, College of Veterinary Medicine and Research Institute for Veterinary Science, Seoul National University, Seoul, South Korea; ^3^ Department of Microbiology, Institute for Immunology and Immunological Diseases, Brain Korea 21 PLUS Project for Medical Science, Yonsei University College of Medicine, Seoul, South Korea

**Keywords:** *Mycobacterium abscessus*, type I IFN, NOD2, nitric oxide, macrophage

## Abstract

*Mycobacterium abscessus* (MAB) is one of the rapidly growing, multidrug-resistant non-tuberculous mycobacteria (NTM) causing various diseases including pulmonary disorder. Although it has been known that type I interferons (IFNs) contribute to host defense against bacterial infections, the role of type I IFNs against MAB infection is still unclear. In the present study, we show that rIFN-β treatment reduced the intracellular growth of MAB in macrophages. Deficiency of IFN-α/β receptor (IFNAR) led to the reduction of nitric oxide (NO) production in MAB-infected macrophages. Consistently, rIFN-β treatment enhanced the expression of iNOS gene and protein, and NO production in response to MAB. We also found that NO is essential for the intracellular growth control of MAB within macrophages in an inhibitor assay using iNOS-deficient cells. In addition, pretreatment of rIFN-β before MAB infection in mice increased production of NO in the lungs at day 1 after infection and promoted the bacterial clearance at day 5. However, when alveolar macrophages were depleted by treatment of clodronate liposome, rIFN-β did not promote the bacterial clearance in the lungs. Moreover, we found that a cytosolic receptor nucleotide-binding oligomerization domain 2 (NOD2) is required for MAB-induced TANK binding kinase 1 (TBK1) phosphorylation and IFN-β gene expression in macrophages. Finally, increase in the bacterial loads caused by reduction of NO levels was reversed by rIFN-β treatment in the lungs of NOD2-deficient mice. Collectively, our findings suggest that type I IFNs act as an intermediator of NOD2-induced NO production in macrophages and thus contribute to host defense against MAB infection.

## Introduction

*Mycobacterium abscessus* (MAB) is one of the rapidly growing non-tuberculous mycobacteria (NTM) causing chronic pulmonary infection and skin and soft tissue infection (SSTI) in immunosuppressed patients (1). MAB has been well-known for being multidrug resistant, and cases of recurrent infection have been reported despite macrolide, amikacin, cefoxitin, and imipenem treatment (2). Recent Canadian studies have shown that the number of patients infected with MAB is increasing every year and about four times higher than the incidence of tuberculosis (3). As an intracellular pathogen, MAB can survive within innate immune cells such as macrophages and escape host immune response. Thus, understanding the exact molecular mechanism of antimicrobial effect within macrophages against MAB infection is essential for prevention and treatment of MAB infection (4).

Type I interferons (IFNs) are well-known cytokines that play an important role in host antiviral responses, and there are 13 different type I IFN subfamilies (5). The genes encoding type I IFNs are located in the same chromosomal locus; and the IFN-β gene, one of the subfamily, is known as a primordial gene of type I IFN family (6). Microbial pathogen or damage-associated molecular pattern (DAMP) stimulates pattern recognition receptor (PRR), and this stimulation produces type I IFNs through TANK binding kinase 1 (TBK1)–IFN regulatory factor (IRF) signaling cascade (7). Consequently, type I IFNs, including IFN-β, is recognized by the IFN-α/β receptor (IFNAR), and this recognition leads to the transcription of various IFN-stimulated gene (ISG) mediating antiviral effects and various immune responses (6). Nucleotide-binding oligomerization domain 2 (NOD2) is a member of cytosolic nucleotide-binding oligomerization domain (NOD)-like receptor family (8). Upon detecting muramyl dipeptide (MDP) or bacterial peptidoglycan component, NOD2 initiates innate immune response against various microbial pathogens (9). Unlike other bacterial pathogens, mycobacteria produce characteristic enzyme called *N*-acetyl muramic acid hydroxylase (NamH) that converts the *N*-acetylated glycan chain of MDP to *N*-glycolylated chain (10). Moreover, *N*-glycolyl MDP exerts higher NOD2 activity than *N*-acetyl MDP and induces more potent immune response (11). For this reason, the essential role of NOD2 in host innate and adaptive immune responses against infections with *Mycobacterium* spp. has been extensively studied (12–15). Furthermore, we previously identified that NOD2 enhances the antimicrobial effect of macrophage against MAB infection by amplifying NO production, through *in vitro* and *in vivo* experiments (16).

Recently, several studies have been reported on the role of type I IFNs in host immune responses against bacterial infection (17). Interestingly, the role of type I IFNs in the aspect of antimicrobial effect is still controversial depending on bacterial species. In the case of systemic infection of *Streptococcus pneumoniae* and *Escherichia coli*, IFNAR-deficient mice maintained higher bacterial load in blood than did wild-type (WT) mice, which means that type I IFN signaling enhances bacterial clearance (18). Similarly, in case of *Helicobacter pylori* oral infection, IFNAR-deficient mice exhibited higher bacterial load in the stomach than did WT mice (19), correlating with the higher mortality in IFNAR-deficient mice in cecal ligation puncture-induced mouse sepsis model (20). On the contrary, IFNAR- or IFN-β-deficient mice exhibited lower bacterial load than did WT mice in the cases of *Francisella tularensis*, *Salmonella typhimurium*, and *Listeria monocytogenes* infection (21), indicating that type I IFNs exerted detrimental effects on host antimicrobial responses. In the case of mycobacterial infection, though belonging to the same genus, each species showed different phenotypes depending on the presence or absence of type I IFN signaling. INFAR-deficient mice infected with *Mycobacterium tuberculosis* exhibited lower bacterial loads in lung than the WT mice (22). On the other hand, the bacterial load of *Mycobacterium avium* complex and *Mycobacterium smegmatis* remained higher in IFNAR-deficient mice than WT mice (23). Even for the studies on *M. tuberculosis* infection, the impact of type I IFNs on bacterial clearance is different from study to study (24). For these reasons, it is important to identify the varying roles of type I IFN in host defense in the context of different experimental conditions such as bacterial species, infection dose, and infection route.

Likewise, the controversial roles of type I IFNs in MAB infection have been reported in two recent publications (25, 26). These two studies provided the conflicting results in antimicrobial responses of macrophages depending on the presence and absence of type I IFN signal. The first study reported that type I IFNs augment the cell-to-cell spread of MAB by increasing cytotoxicity of infected macrophages resulting in IFNAR-deficient macrophages that exhibited lower bacterial load as compared with the WT macrophages (26). The other reported that type I IFNs increased the production of NO in MAB-infected macrophages and that, consequently, IFNAR-deficient macrophages showed higher bacterial load than the WT macrophages in their study (25). Furthermore, there has been no study that elucidates the role of type I IFNs in *in vivo* model of MAB infection.

In this study, we aimed to i) clarify the antimicrobial mechanism of type I IFNs during MAB infection in macrophages and ii) elucidate the role of type I IFNs in *in vivo* MAB pulmonary infection model.

## Materials and Methods

### Cell Culture and Medium

Bone marrow-derived macrophages (BMDMs) were derived from murine femur–tibia bone marrow and prepared as previously described (27). In summary, isolated BMDMs were incubated at 5% CO_2_, 37°C in complete Iscove’s modified Dulbecco’s medium (IMDM; Gibco, Grand Island, NY, USA) supplemented with 12.5 ng/ml of recombinant mouse M-CSF Protein (R&D Systems, Minneapolis, MN, USA), 10% fetal bovine serum (FBS), 1% sodium pyruvate, 1% MEM Non-Essential Amino Acids (MEM NEAA), and 1% penicillin/streptomycin (P/S). After 3 days, 5 ml of fresh same medium was added, and the cells were cultured under the same conditions for 3 days and then used in the experiment. For the treatment of bacteria and reagents, medium (hereafter referred to as treat medium) was supplied to IMDM with 2% FBS, 1% MEM NEAA, and 1% sodium pyruvate without antibiotics.

MH-S mouse alveolar macrophage (AM) cells purchased from American Type Culture Collection (ATCC, Manassas, VA, USA) were cultured in Roswell Park Memorial Institute (RPMI) 1640 (WELGENE, Gyeongsan, Republic of Korea) supplemented with 10% FBS, 50 μM of 2-mercaptoethanol, and 1% P/S at 37°C in 5% CO_2_ conditions. Treat medium was supplied to RPMI 1640 with 2% FBS and 50 μM of 2-mercaptoethanol without antibiotics.

Murine primary AMs were cultured as previously described (28). Briefly, 8- to 15-week-old male mice with C57BL/7 background were anesthetized, and bronchoalveolar lavage fluid (BALF) was obtained by flushing 1 ml of cold Dulbecco’s phosphate-buffered saline (D-PBS, WELGENE, Gyeongsan, Korea) containing 1 mM of EDTA. Collected BALFs from each mouse were pooled and centrifuged with 4°C and 250 relative centrifugal force (RCF). After the supernatant was discarded, pellets were dissolved with red blood cell (RBC) lysis buffer and incubated at room temperature for 10 min. After being washed twice with D-PBS, AMs were dissolved with DMEM high-glucose medium containing 10% FBS, 1% P/S, 1% HEPES, and 1% sodium pyruvate. AMs were seeded on a 48-well plate, 200 μl per well, with a concentration of 5 × 10^5^ cell/ml. After 12 h, the time to stabilize cells after attaching to plate, intracellular bacterial growth assay was performed with the same method of BMDMs and MH-S cells. Treat medium was supplied to DMEM high-glucose medium with 2% FBS, 1% HEPES, and 1% sodium pyruvate without antibiotics.

### Reagents

The following reagents were used. Mouse recombinant IFN-β (PBL Biomedical Laboratories, Piscataway, NJ, USA; Cat# 12401-1). Ultrapure lipopolysaccharide from *E. coli* O111:B4 (LPS; InvivoGen, San Diego, CA; Cat# tlrl-eblps), *N* ^G^-Nitro-l-arginine methyl ester (l-NAME; Sigma Aldrich, St. Louis, MO, USA, Cat# 51298-62-5), and BX795 (InvivoGen, San Diego, CA, USA, Cat# tlrl-bx7).

### Mice

WT, *Ifnar* ^−/−^, *Inos* ^−/−^, and *Nod2* ^−/−^ mice on a C57BL/6 background were purchased from The Jackson Laboratory (Bar Harbor, ME, USA). Female mice aged 8–10 weeks were used for animal experiments. Animal experiments were performed under protocols approved by the Institutional Animal Care and the Use Committee of Chonnam National University (Approval No. CNU IACUC-YB-2017-56 and 2019-31).

### Bacterial Culture and *In Vitro* Infection Dose

Bacterial culture of the isogenic rough variant of *M. abscessus* ATCC19977T (Manassas, VA, USA) were prepared as previously described (29). Briefly, the bacterium was cultivated in 7H9 broth supplemented with 0.5% glycerol, 10% oleic acid, dextrose, albumin, and catalase (OADC; BD Biosciences, San Jose, CA, USA). For seed culture, 1 ml of frozen bacterial stock (3 × 10^8^ CFU) was added in 10 ml of culture medium, and the cells were allowed to grow for 7 days at 37°C with shaking at 130 rpm. For main culture, the entire cells from seed culture were added to 200 ml of culture medium and incubated for three more days. The cells were centrifuged at 4,500 RCF and washed three times with D-PBS. For single-cell suspension, the cells were dissolved in D-PBS and sonicated for 1 s at 40 kHz, 100 W. The cells aggregated by sonication were removed by passing through 40-μm mesh. This process was repeated three times. The seed lots dissolved in D-PBS (contained 40% glycerol) were kept at −80°C until use. In all *in vitro* experiments, the number of macrophages to the number of bacteria was set as 1:25 multiplicity of infection (MOI). This infection dose was determined in our previous study (16). Except the assessment of TBK1 phosphorylation (Western blotting), extracellular MAB was washed out after 1 h post infection, and cells were incubated with gentamicin (Sigma Aldrich, St. Louis, MO, USA) containing fresh medium for indicated times in all experiments

### Intracellular Bacterial Growth Assay and Colony-Forming Unit Measurement

BMDMs and MH-S cells were seeded on a 48-well plate, 200 μl per well, with a concentration of 1 × 10^6^ cell/ml. After 12 h, the time to stabilize cells after attaching to plate, MAB diluted in treat medium was infected for 200 μl per well with MOI 25. After 1 h, which is a sufficient time for phagocytosis, cell culture supernatant was washed out by D-PBS and replaced with fresh treated medium containing 100 μg/ml of gentamicin. Cell culture supernatant was washed out by D-PBS on indicated time, and attached cells were lysed with 100 μl of 1% Triton X-100. The bacterial colony-forming unit (CFU) of cell lysate was determined by the spread plate technique, as follows. The cell lysate or lung lysate (*in vivo*) samples containing bacteria were diluted with D-PBS as proper detectable levels. Fifty microliters of diluted samples was uniformly spread through glass beads on the Middlebrook 7H10 agar (Difco, Detroit, MI, USA) plate containing ampicillin, and plates were incubated for 96 h at 37°C. The number of colony was counted and converted to log value. The CFU value of the tissue sample was normalized to tissue weight (g).

### Real-Time Quantitative Polymerase Chain Reaction

BMDMs and MH-S cells were seeded on a 6-well plate, 2 ml per well, with a concentration of 1 × 10^6^ cell/ml. After 12 h, the time to stabilize cells after attaching to plate, infection and reagent treatment were performed with each indicated conditions. RNA was extracted using 1 ml of easy-BLUETM Total RNA Extraction Kit (iNtRON Biotechnology, Seongnam, Korea). cDNA was synthesized from 1 μg of RNA using ReverTra Ace^®^ qPCR RT Master Mix (TOYOBO Biotechnology, Osaka, Japan). Real-time qPCR samples were prepared using the SYBR Green PCR Kit (Qiagen GmbH, Hilden, Germany). The primer sequences were as follows: iNOS; sense, 5′-GCATTGGAAGTGAAGCGTTTC-3′ and antisense, 5′-GGCAGCCTGTGAGACCTTTG-3′. IFN-β; sense 5′-ATGAACTCCACCAGCAGACAG-3′, and antisense, 5′-ACCACCATCCAGGCGTAGC-3′. CRAMP; sense 5′-AAGGAACAGGGGGTGGTG-3′ and antisense, 5′-CCGGGAAATTTTCTTGAACC-3′. NOX2; sense 5′-ACTCCTTGGAGCACTGG and antisense 5′-GTTCCTGTCCAGTTGTCTTC-3′. β-Actin; sense 5′-AGGCCCAGAGCAAGAGAG-3′ and antisense, 5′-TCAACATGATCTGGGTCAT-3′. PCR was conducted by a Rotor-Gene Q real-time PCR system (Qiagen) using a two-step protocol of 40 cycles of 95°C for 10 s followed by 60°C for 45 s. Normalized gene expression levels were indicated as the ratio between the mean value for the target gene and that for the β-actin.

### Western Blotting

BMDMs were seeded on a 12-well plate, 1 ml per well, with a concentration of 1 × 10^6^ cell/ml. After 12 h, the time to stabilize cells after attaching to plate, infection and reagent treatment were performed with each indicated conditions. Cells were lysed using lysis buffer containing Nonidet P‐40, complete protease inhibitor cocktail (Roche, Mannheim, Germany), and 2 mM of dithiothreitol. Cell lysates were separated using sodium dodecyl sulfate–polyacrylamide gel electrophoresis (SDS-PAGE) and transferred to nitrocellulose membranes. The membranes were incubated at 4°C with anti-iNOS (1:2,000 dilution) (BD Biosciences, San Jose, CA, USA), anti-phospho-TBK1 (1:1,000 dilution) (Cell Signaling Technology, Beverly, MA, USA), anti-total-TBK1 (1:1,000 dilution) (Cell Signaling Technology, Beverly, MA, USA), and anti‐β‐actin (1:2,000 dilution) (Santa Cruz Biotechnology, Santa Cruz, CA) for 24 h. After the primary antibody is attached, the relevant secondary antibodies were attached at room temperature for 2 h. Proteins were detected by an enhanced chemiluminescence reagent (iNtRON Biotechnology, Seongnam, Korea).

### Measurement of Nitric Oxide Levels in Cell Culture Supernatants

BMDMs and MH-S cells were seeded on a 48-well plate, 200 μl per well, with a concentration of 1 × 10^6^ cell/ml. After bacterial infection and reagent treatment according to each condition, cell culture supernatants were harvested, and NO concentrations were measured *via* the Griess reaction assay as previously described (30).

### Enzyme-Linked Immunosorbent Assay

To investigate the difference in inflammatory cytokine production depending on the presence or absence of type I IFN signal in the lung of MAB-infected mice, IL-6 (assay range 15.6–1,000 pg/ml), TNF-α (assay range 31.2–2,000 pg/ml), IL-10 (assay range 31.2–2,000 pg/ml), and IL-1β (assay range 15.6–1,000 pg/ml) cytokines from the supernatant of lung lysate were measured by ELISA kits (R&D System, Minneapolis, MN, USA) according to the manufacturer’s instructions. The cytokine value of the tissue sample was normalized to tissue weight (g).

### Lactate Dehydrogenase Assay

To elucidate the difference in cytotoxicity depending on the presence or absence of type I IFN signal in the MAB infection, BMDMs and MH-S cells were seeded on a 48-well plate, 200 μl per well, with a concentration of 1 × 10^6^ cell/ml. After bacterial infection and reagent treatment according to each condition, cell culture supernatant was harvested, and lactate dehydrogenase (LDH) levels were measured by CytoTox 96^®^ Non-Radioactive Cytotoxicity Assay kits (Promega, Madison, WI, USA) according to the manufacturer’s instructions.

### Mouse Infection Model

For intranasal inoculation or administration, mice were anesthetized with combination of xylazine (10 mg/kg) and zoletil (30 mg/kg). Mice were inoculated with 2 × 10^7^ CFU (in 40 μl of D-PBS) of MAB intranasally. Eight hundred units (in 40 μl of D-PBS) per mouse of rIFN-β was administered in the same way as bacterial inoculation. At indicated day post infection, mice were sacrificed, and lung tissues were harvested in a sterilized condition. The rest of the lobes except the left lobe were homogenized with 500 μl of D-PBS, and the lysate was used for CFU measurements. The remaining lysate was centrifuged for 10 min at 4,000 RCF, and the supernatant was harvested for NO measurement and cytokine analysis. NO concentrations were measured by the Griess reaction assay, and cytokines were analyzed by ELISA. The left lung lobe was used for the histological examination.

### Clodronate Liposomes and Macrophage Depletion

Clodronate liposomes and control liposomes were purchased from LIPOSOMA (Amsterdam, Netherlands, Cat# CP-005-005). Animal experiments for the depletion of macrophages in the lungs were performed as follows: 50 μl of clodronate liposomes or control liposomes was administrated intranasally under anesthesia once a day for 3 days. The administration of rIFN-β and bacterial infection were performed according to the schedule indicated in diagram of result section.

### Flow Cytometry

The left lobes of the lung tissue were pooled by each group and slightly chopped using a pair of scissors in cold RPMI 1640 containing 1 μg/ml of dipase II (Sigma Aldrich, St. Louis, MO, USA). Samples were incubated at 37°C, 1,100 RPM shaking chamber. After 1 h, samples were placed on 40-μm cell strainer and homogenized using syringe rubber to obtain the single cells. RBCs were lysed using RBC lysis buffer, and flow cytometry assay was performed as previously described (31). In briefly, cells were stained with anti-CD45-APC (BD Biosciences, San Jose, CA, USA), anti-CD11c-PE (BD Biosciences), and anti-F4/80-FITC (Invitrogen, Carlsbad, CA, USA). Analysis was performed by using MACSQuant Analyzer 10 (Miltenyi Biotec, Bergisch Gladbach, Germany).

### Histopathological Examination

The left lobe of the lung was fixed in 10% neutral formalin for 24 h, followed by tissue processing and paraffin embedding. The paraffin blocks were sectioned at 2 μm and stained with H&E. Histopathological examination was performed under microscopy. Histopathological severity was scored blindly by two experts in a field of laboratory animal pathology with an arbitrary scoring index based on the degree of inflammatory cell infiltration and the extent of the lesion area (0, normal; 1, mild; 2, mild to moderate; 3, moderate; 4, moderate to severe; and 5, severe).

### Statistical Analysis

The statistical significance of differences between two groups was determined by unpaired t-test. In case of more than three groups, the statistical significance was determined by one-way ANOVA followed by Tukey’s post-hoc test for comparisons between groups (GraphPad Prism 5; GraphPad Software Inc., La Jolla, CA, USA). p-Values <0.05 were considered statistically significant.

## Results

### *Mycobacterium abscessus* Induces Interferon-β Gene Expression of Macrophages in TANK Binding Kinase 1-Dependent Manner, Which Promotes Intracellular Bacterial Clearance

**Figure 1:**
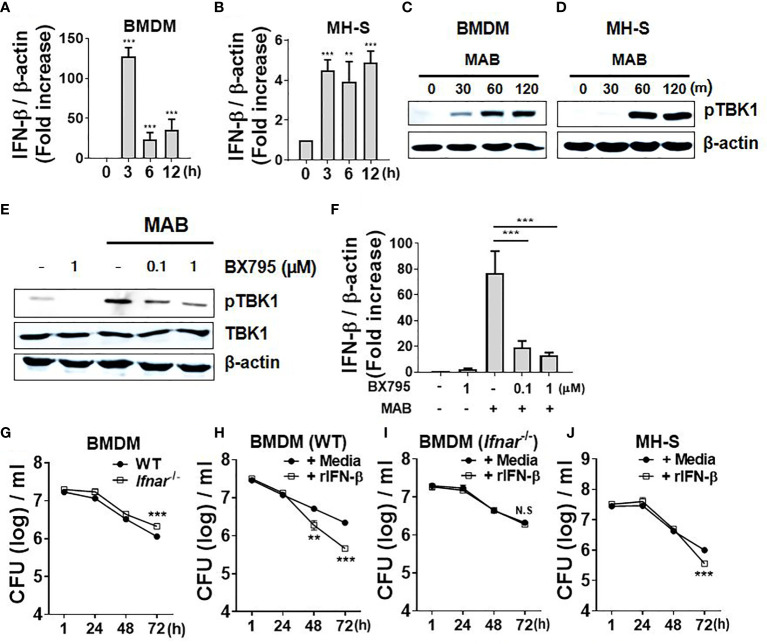
MAB induces IFN-β gene expression of macrophages in TBK1-dependent manner, which promotes intracellular bacterial clearance. **(A, B)** BMDMs and mouse alveolar macrophage cell line MH-S cells were infected with MAB at multiplicity of infection (MOI) 1:25 for indicated times. mRNA was extracted, and the expression levels of IFN-β gene were determined by real-time PCR. **(C)** BMDMs and **(D)** MH-S cells were infected with MAB for indicated times. **(E)** BMDMs were infected with MAB for 1 h in the absence or presence of BX795 (2 h pretreated). **(C–E)** The levels of indicated proteins were determined by Western blotting. **(F)** BMDMs were infected with MAB for 6 h in the absence or presence of BX795 (2 h pretreated). The mRNA was extracted, and the expression levels of IFN-β gene were determined by real-time PCR. **(G–J)** Cells were infected with MAB in the absence or presence of recombinant IFN-β (1,000 units/ml, 2 h pretreated). Intracellular bacterial CFU on indicated times were evaluated by intracellular bacterial growth assay. **(A–J)** The results are from one representative experiment of two independent experiments (**p < 0.01, ***p < 0.001). MAB, *Mycobacterium abscessus*; IFN, interferon; TBK1, TANK binding kinase 1; BMDMs, bone marrow-derived macrophages; CFU, colony-forming unit. NS, Not Statistically Significant.

We first examined whether MAB induces gene expression of type I IFNs in macrophages. Consistent with previous studies (25, 26), MAB upregulated IFN-β gene expression approximately 125-fold compared with 0 h in BMDMs as well as fivefold in MH-S cells, a murine AM cell line, at 3 h after infection (**Figures 1A, B**). Various PRR signaling mediates type I IFN expression by activating TBK1 (5). As expected, infection with MAB induced phosphorylation of TBK1 in both BMDMs and MH-S cells (**Figures 1C, D**). Pretreatment with BX795, a TBK1-specific inhibitor, suppressed MAB-induced TBK1 phosphorylation and IFN-β gene expression in a dose-dependent manner in BMDMs (**Figures 1E, F**), indicating that MAB induces gene expression of type I IFNs in macrophages *via* TBK1-dependent manner.

There has been an extreme controversy on the role of type I IFNs in the intracellular survival of MAB within macrophages (25, 26). To clarify this, we examined the intracellular bacterial CFUs under various experimental conditions. Compared with WT BMDMs (6.05 log CFU/ml), the bacterial CFUs were higher in IFNAR-deficient BMDMs (6.32 log CFU/ml) at 72 h after infection (**Figure 1G**). In addition, treatment of recombinant IFN-β (rIFN-β) enhanced the bacterial clearance from 6.34 log CFU/ml to 5.66 log CFU/ml in WT BMDMs, but not in IFNAR-deficient cells (**Figures 1H, I**) at 72 h after infection. In MH-S cells, the bacterial clearance was accelerated by rIFN-β from 6 log CFU/ml to 5.55 log CFU/ml at 72 h after infection (**Figure 1J**). These results suggest that type I IFNs inhibit the intracellular growth of MAB in macrophages.

### Type I Interferons Augment *Mycobacterium abscessus*-Induced Production of Nitric Oxide in Macrophages

**Figure 2:**
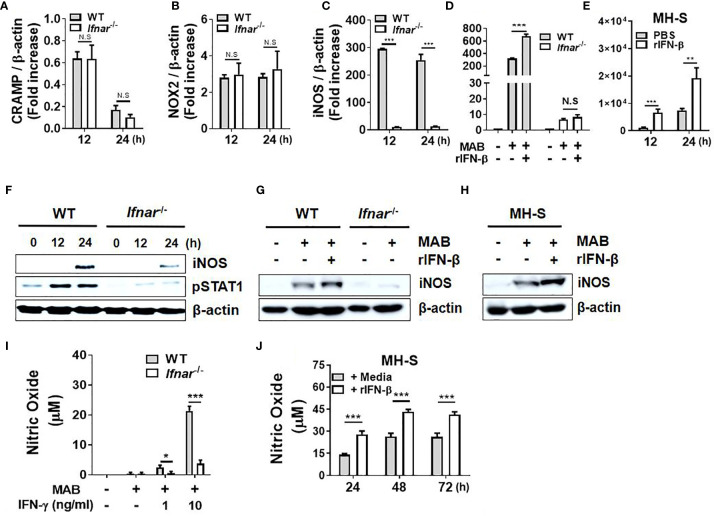
Type I IFNs augment MAB-induced production of nitric oxide in macrophages. **(A–C)** WT and IFNAR-deficient BMDMs were infected with MAB at a MOI 1:25 for indicated times. **(D, E)** BMDMs and MH-S cells were pretreated with or without rIFN-β (1,000 units/ml) for 2 h and additionally infected with MAB for **(D)** 12 h or **(E)** indicated times. **(A–E)** mRNA was extracted, and the expression levels of each gene were determined by real-time PCR. **(F)** BMDMs were infected with MAB for indicated times. **(G)** BMDMs and **(H, J)** MH-S cells were pretreated with or without rIFN-β (1,000 units/ml) for 2 h and additionally infected with MAB for **(G, H)** 24 h or **(J)** indicated times. **(I)** BMDMs were incubated for 24 h with indicated conditions. **(F–H)** Cellular proteins were extracted, and the levels of indicated proteins were determined by Western blotting. **(I, J)** Nitric oxide concentration in cell culture supernatant was measured by Griess reaction. **(A–J)** The results are from one representative experiment of two independent experiments (*p < 0.05, **p < 0.01, ***p < 0.001). MAB, *Mycobacterium abscessus*; IFN, interferon; WT, wild type; IFNAR, interferon-α/β receptor; BMDMs, bone marrow-derived macrophages; MOI, multiplicity of infection. NS, Not Statistically Significant.

Antimicrobial peptides (AMPs), reactive oxygen species (ROS), and NO are the major factors involved in the removal of intracellular pathogens in macrophages (32). We sought to determine whether type I IFNs regulate expression of those factors in response to MAB in macrophages. IFNAR deficiency did not affect the gene expression of CRAMP and NOX2 (**Figures 2A, B**), whereas iNOS expression was mostly abolished in IFNAR-deficient BMDMs (**Figure 2C**). Treatment with rIFN-β also enhanced MAB-induced iNOS expression in WT BMDMs, but not in IFNAR-deficient BMDMs (**Figure 2D**). Moreover, rIFN-β also increased MAB-induced iNOS expression in MH-S cell (**Figure 2E**). The protein expression of iNOS induced by MAB was reduced in IFNAR-deficient macrophages *versus* the WT cells (**Figure 2F**), and rIFN-β enhanced MAB-induced expression of iNOS protein in BMDMs (**Figure 2G**) and MH-S cells (**Figure 2H**), but not in IFNAR-deficient BMDMs (**Figure 2G**). Our previous study revealed that MAB alone could not produce detectable level of NO in BMDMs, but at the presence of the type II IFN, IFN-γ, the bacterium led to substantial level of NO production (16). Consistently, at the presence of IFN-γ, MAB induced NO production in WT BMDMs, which were mostly abolished in IFNAR-deficient cells (**Figure 2I**). Unlike BMDMs, MAB alone could produce detectable levels of NO in MH-S cells, which were also enhanced by rIFN-β (**Figure 2J**). These results suggest that type I IFN signaling may contribute to MAB-induced NO production in macrophages.

### Type I Interferons Inhibit the Intracellular *Mycobacterium abscessus* Growth in Macrophages by Regulating Nitric Oxide Production

**Figure 3:**
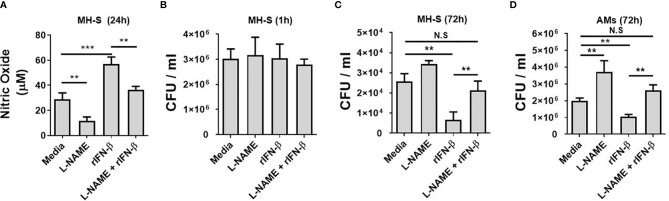
Type I IFNs inhibits the intracellular MAB growth in macrophages by regulating NO production. **(A–C)** MH-S cells were incubated with MAB and indicated reagents (2 h pretreated). **(A)** Nitric oxide concentration in cell culture supernatant was measured by Griess reaction at 24 h post infection. Intracellular bacterial CFU on **(B)** 1 h and **(C)** 72 h were evaluated by intracellular bacterial growth assay. **(D)** AMs were incubated with MAB (MOI 1:25) and indicated reagents (2 h pretreated, l-NAME 1 mM, rIFN-β 1,000 units/ml). Intracellular bacterial CFU on 72 h was evaluated by intracellular bacterial growth assay. **(A–D)** The results are from one representative experiment of two independent experiments (**p < 0.01, ***p < 0.001). MAB, *Mycobacterium abscessus*; IFN, interferon; CFU, colony-forming unit; AMs, alveolar macrophages; MOI, multiplicity of infection. NS, Not Statistically Significant.

It has been reported that NO is critical for the restriction of intracellular MAB growth in macrophages (16, 25, 33). As type I IFNs enhanced the intracellular MAB clearance and MAB-induced NO production in BMDMs at the presence of IFN-γ and MH-S cells, we sought to clarify whether NO is a key factor for type I IFN-mediated inhibition of the bacterial growth. In the presence of l-NAME, a non-selective nitric oxide synthase (NOS) inhibitor, there was no significant difference in the bacterial CFUs between WT and IFNAR-deficient BMDMs (**Supplementary Figure 1A**). Treatment of rIFN-β also did not affect the intracellular growth of MAB in l-NAME-treated or iNOS-deficient BMDMs (**Supplementary Figures 1B, C**). As shown in **Figure 3A**, l-NAME reduced MAB-induced or rIFN-β-enhanced NO production in MH-S cells. Treatment with l-NAME or rIFN-β did not affect the phagocytosis of MH-S cells (**Figure 3B**). Consistent with the result presented in **Figure 1J**, rIFN-β inhibited the MAB growth 3.8-fold compared with medium in MH-S cells, which was restored by l-NAME treatment (**Figure 3C**). This phenomenon was also confirmed in primary isolated murine AMs. The intracellular growth of MAB was 1.86-fold higher in l-NAME-treated AMs compared with medium-treated AMs at 72 h post infection (**Figure 3D**). Also, while rIFN-β inhibited the growth of MAB 1.91-fold compared with medium control, in the presence of l-NAME, rIFN-β did not suppress the intracellular growth of MAB in AMs, 72 h after infection (**Figure 3D**). These results indicate that type I IFNs suppress the intracellular survival of MAB by promoting NO production in macrophages.

### Intranasal Pretreatment of rIFN-β Augments Nitric Oxide Production and Promotes the *Mycobacterium abscessus* Clearance in the Lungs of Mice

**Figure 4:**
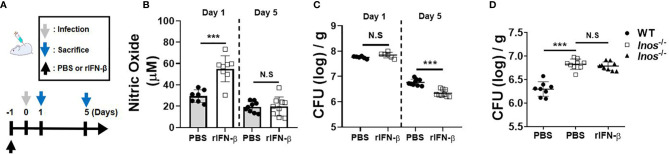
Intranasal pretreatment of rIFN-β augments NO production and promotes the bacterial clearance in the lungs of mice infected with MAB. **(A)** The administration of rIFN-β and bacterial infection were performed according to the schedule indicated in the diagram. **(B–D)** WT or iNOS-deficient mice were administrated with PBS or rIFN-β (800 units per mouse) intranasally under anesthesia. After 1 day, mice were infected with 2 × 10^7^ CFU of MAB per mouse intranasally; and the bacterial loads and nitric oxide levels in the lung lysate were determined at indicated days. **(B–D)** The results are merged data of two independent experiments (n = 4–6) (***p < 0.001). MAB, *Mycobacterium abscessus*; WT, wild type; PBS, phosphate-buffered saline; CFU, colony-forming unit. NS, Not Statistically Significant.

Next, we sought to determine *in vivo* role of type I IFNs against pulmonary infection of MAB by pre-exposure to rIFN-β. The experimental schedule is depicted in **Figure 4A**. NO levels in the lung homogenate of mice pretreated (i.n.) with rIFN-β were higher than those of the mice treated with PBS at day 1 after infection, although the difference between the two groups was not observed at day 5 (**Figure 4B**). Pretreatment of rIFN-β also reduced the bacterial CFUs in the lung homogenates of MAB-infected mice at day 5, but not at day 1 (**Figure 4C**). To determine the *in vivo* effect of type I IFN-induced NO on the growth control of MAB, we repeated the experiment using iNOS-deficient mice. As presented in **Figure 4D**, intranasal pretreatment with rIFN-β did not influence the MAB growth in the lungs of iNOS-deficient mice (**Figure 4D**), strongly supporting the notion that intranasal pre-exposure to rIFN-β contributes to *in vivo* growth control of MAB by promoting NO production in macrophages. However, administration of rIFN-β after MAB infection did not improve the bacterial clearance in the lungs of MAB-infected mice (**Supplementary Figures 2A, B**). To determine the impact of type I IFNs on host defense against MAB infection, we compared the bacterial CFUs in the lungs of WT and IFNAR-deficient mice. Unexpectedly, the bacterial CFUs at day 5 were significantly lower in IFNAR-deficient mice as compared with WT mice (**Supplementary Figures 3A, B**), suggesting that type I IFNs can exert a harmful effect in host defense against MAB infection, although prophylactic administration of type I IFNs improves *in vivo* growth control of MAB.

### Macrophages Are Responsible for rIFN-β Response in *Mycobacterium abscessus*-Induced Nitric Oxide Production and the Bacterial Clearance *In Vivo*

**Figure 5:**
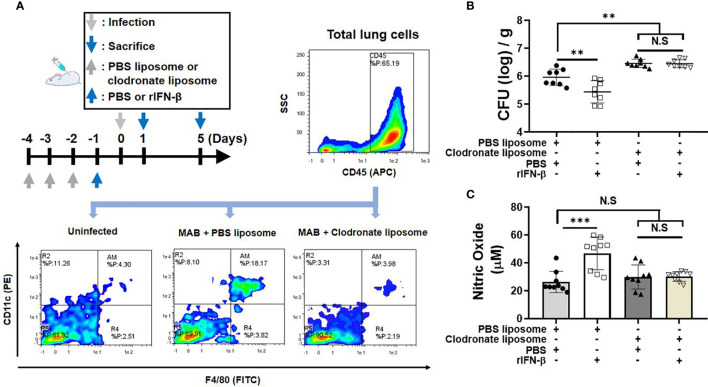
Macrophages are responsible for rIFN-β response in MAB-induced NO production and the bacterial clearance *in vivo*. **(A)** The administration of reagents and bacterial infection were performed according to the schedule indicated in the diagram. Flow cytometric plot showing the population of CD45^+^CD11c^+^F4/80^+^ alveolar macrophage in lung total cell at 1 day post infection. **(B)** Mice were administrated with indicated reagents intranasally under anesthesia. After 1 day, mice were infected with 2 × 10^7^ CFU of MAB per mouse intranasally, and the bacterial loads in the lung lysate were determined at 5 days post infection. **(C)** Nitric oxide levels were measured on 1 day post infection in the lung lysate supernatant. **(A)** The results are one representative data of two independent experiments (n = 4). **(B, C)** The results are merged data of two independent experiments (n = 4–5) (**p < 0.01, ***p < 0.001). MAB, *Mycobacterium abscessus*; CFU, colony-forming unit. NS, Not Statistically Significant.

We investigated whether macrophages are involved in the *in vivo* protective effect of rIFN-β against the pulmonary infection of MAB. Schedule of clodronate injection and MAB infection is depicted in **Figure 5A**. The bacterial CFUs in the lungs were examined at day 5 after infection and NO production at day 1. Flow cytometry analysis showed that MAB infection increased the ratio of the CD45^+^F4/80^+^CD11c^+^ AM in the lungs from 4% to 18%, which was rescued to 4% by the treatment with clodronate liposome (**Figure 5A**). In mice treated with clodronate, rIFN-β treatment did not suppress the bacterial growth in the lungs, whereas the bacterial CFUs were decreased by rIFN-β treatment in mice with PBS liposome (**Figure 5B**). Moreover, rIFN-β treatment could not elicit NO production in mice treated with clodronate liposome (**Figure 5C**). Based on these data, it is likely that macrophages are essential for the protective effect of rIFN-β against MAB infection.

### Type I Interferons Act as an Intermediator in a Cytosolic Receptor NOD2-Mediated Nitric Oxide Production in Response to *Mycobacterium abscessus*

**Figure 6:**
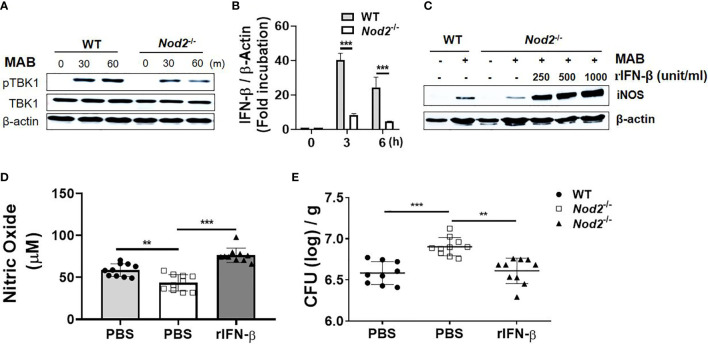
Type I IFNs act as an intermediator of a cytosolic receptor NOD2-mediated NO production in response to MAB. **(A)** BMDMs were infected with MAB for indicated times. Cellular proteins were extracted, and the levels of each protein were determined by Western blotting. **(B)** BMDMs were infected with MAB for indicated time. mRNA was extracted, and the expression levels of IFN-β gene were determined by real-time PCR. **(C)** BMDMs were incubated for 24 h with indicated conditions. The levels of each protein were determined by Western blotting. **(D)** Nitric oxide levels were measured on 1 day post infection in the lung lysate (same conditions with **Figure 4**). **(E)** The bacterial load in the lung lysate was determined at 5 days post infection. **(A–C)** The results are from one representative experiment of two independent experiments. **(D, E)** The results are merged data of two independent experiments (n = 5) (**p < 0.01, ***p < 0.001). MAB, *Mycobacterium abscessus*; IFN, interferon; BMDMs, bone marrow-derived macrophages.

Our previous study demonstrated that NOD2 contributes to *in vivo* and *in vitro* bacterial clearance against MAB infection by promoting NO production (16). In addition, Pandey et al. reported that NOD2-Ripk2 signaling is involved in induction of type I IFNs against *M. tuberculosis* infection through bacterial ESX-1- and host TBK1-IRFs-dependent pathways (34). Thus, we hypothesized that NOD2 mediates MAB-induced type I IFN expressions, which results in NO-mediated killing of MAB. NOD2 deficiency diminished MAB-induced phosphorylation of TBK1 in BMDMs (**Figure 6A**). In addition, the gene expression of IFN-β in response to MAB was significantly reduced in NOD2-deficient BMDMs as compared with WT cells (**Figure 6B**). The protein expression of iNOS was also decreased in NOD2-deficient BMDMs, which was restored by the addition of rIFN-β (**Figure 6C**). In an *in vivo* experiment, MAB-induced NO production was decreased in NOD2-deficient mice at day 1, which was also restored by rIFN-β (**Figure 6D**). The bacterial clearance in the lungs was impaired in NOD2-deficient mice at day 5 (**Figure 6E**). However, intranasal administration of rIFN-β reduced the bacterial CFUs in the lungs of NOD2-deficient mice, exhibiting comparable levels of CFUs as in WT mice (**Figure 6E**). These results suggest that NOD2 signaling contributes to the clearance of MAB by promoting type I IFN-mediated NO production in macrophages.

## Discussion

Macrophages play an essential role in host innate immune responses against intracellular bacterial infection (32). In the current investigation, we examined the impact of type I IFNs on bactericidal activity of macrophages and confirmed that IFNAR deficiency causes attenuation of bacterial clearance in macrophages under MAB infection. Moreover, pretreatment of mice with rIFN-β enhanced the bactericidal activity of macrophages. In fact, to evaluate the dose dependence of rIFN-β on MAB growth control, we used two doses of rIFN-β (500 and 1,000 units/ml). However, the 500 units/ml of rIFN-β inhibited the MAB growth at the same level of 1,000 units/ml in BMDMs (data not shown). As shown in **Figure 6C**, rIFN-β strongly increased iNOS protein expression even at a low dose (250 units/ml). Therefore, to see a dose-dependent response of rIFN-β on MAB growth inhibition, it seems that an experiment using much lower concentrations (below 250 units/ml) should be performed. In addition, our results suggested that type I IFNs promote production of NO against MAB infection and that MAB clearance is facilitated in a NO-dependent manner within macrophages. These results are consistent with the previous finding of the role of type I IFNs in *M. tuberculosis*-infected macrophages (35). Interestingly, we found that type I IFN signaling engaged in the robust production of NO at the early stage of MAB infection. However, the difference in intracellular bacterial CFUs depending on the presence or absence of type I IFN signaling was observed at least 48 h post infection. These results are in line with previous studies (16) that showed bactericidal activity of macrophage by NO production after at least 48-h mycobacterial infection. This phenomenon may be related to the virulence mechanisms of *Mycobacterium* spp., which survive within phagosome as an immune evasion strategy (36).

Recently, controversial and complex roles of type I IFNs in MAB-infected macrophages have been reported from different research groups. MAB can lead to various types of cell death such as necrosis, apoptosis, and pyroptosis in macrophages (37–39). Previous studies revealed that rough morphotype of MAB (MAB-R), lacking cell surface glycopeptidolipids, is more proapoptotic than smooth morphotype (MAB-S) and releases more LDH (26, 38, 39). In addition, Kim et al. reported that type I IFNs augment the cell-to-cell spread of MAB by increasing cytotoxicity of infected macrophages (26). As a result, IFNAR-deficient macrophages exhibited lower levels of bacterial load compared with the WT macrophages (26). In this study, we also evaluated the effect of type I IFNs on cytotoxicity of MAB-infected macrophages by measuring LDH release. There was no significant difference in LDH release between the WT and IFNAR-deficient BMDMs in response to MAB (**Supplementary Figure 4A**) and pretreatment of the cells with rIFN-β slightly increased during the release of LDH only in WT BMDMs at 72 h post infection, but not in MH-S cells (**Supplementary Figure 4B**). Consistent with the results from our laboratory, Ruangkiattikul et al. reported that type I IFNs increased the production of NO in MAB-infected macrophages (25). Consequently, IFNAR-deficient macrophages showed higher bacterial load than the WT macrophages in their study (25). In this study, we confirmed that NO production is clearly amplified by type I IFNs, which correlated with the enhancement of bactericidal activity in MAB-infected macrophages.

Although the role of type I IFNs in MAB infection has been reported in several previous publications that utilized *in vitro* assays, none of these studies evaluated the role of type I IFNs in animal models (25, 26, 38). In the current study, the host defensive effect of type I IFNs against MAB pulmonary infection in mouse model was evaluated. Since administration of rIFN-β at 1 or 5 days post infection did not affect the bacterial load in the lungs, we established a model that employed administration of rIFN-β at 1 day before infection. Correlating with the *in vitro* results, rIFN-β-administered mice exhibited deceased bacterial loads in lung lysate samples compared with the PBS-administered control mice at 5 days post infection. Administration of rIFN-β also augmented production of NO in lung samples of MAB-infected mice at 1 day post infection. This host defensive effect of type I IFNs against MAB infection in mice was established in a NO-dependent manner evidenced by the inability to enhance bacterial clearance following rIFN-β administration in iNOS-deficient mice. There was no difference in NO levels between rIFN-β and PBS-administered mice at 5 days post infection, and this is presumably due to the compensation mechanisms of immune homeostasis system such as anti-inflammatory response-mediated downward standardization (40–42).

From the perspective of host defense immune system against bacterial infection, enhancement of immune response can lead to fatal damage in host tissue (43). Hence, histological assessment was performed on lung samples from the MAB-infected mice. Although there was no significant difference in histological assessment *via* H&E staining (**Supplementary Figures 5D, E**), lung inflammatory cytokine levels were slightly diminished in rIFN-β-administered mice (**Supplementary Figures 5A–C**). It is presumed that the diminished lung bacterial load leads to the alleviation in the production of inflammatory cytokines.

Next, we confirmed that clodronate-induced macrophage depletion leads to the increase in the susceptibility to MAB pulmonary infection in mice. Moreover, rIFN-β did not enhance the bacterial clearance nor the production of NO within clodronate-administered mice. These findings suggest that the increased bactericidal activity by type I IFN–NO axis in AM is crucial for the host defense innate immune system against MAB infection. In fact, as significant efficacy was verified in several clinical trials (44–47), NO is a crucial host defense factor against MAB pulmonary infection in humans. Our results suggest that the administration of exogenous type I IFNs, at the early stage of infection, could be considered as a therapeutic candidate for MAB-infected patients. Meanwhile, interstitial macrophages as well as AMs are present in the lung tissue and exhibit various biological functions (48). According to previous reports, administration of clodronate liposomes does not eliminate interstitial macrophages (49, 50). In addition, it is known that interstitial macrophages exist with a lower proportion than AMs in the lung parenchyma and that their ability to produce ROS and NO is also much lower than that of AMs (51). Accordingly, the role of interstitial macrophages is not expected to be significant in type I IFN-induced NO production in the lungs, although we did not check the change of the population of interstitial macrophages by clodronate liposomes.

In contrast to our results, several previous studies reported that type I IFNs negatively affect *in vivo* antimicrobial response against most of intracellular bacterial infection (17). Indeed, we also verified the difference in bacterial loads in lung samples between WT and IFNAR-deficient mice. Interestingly, inconsistent with the observed phenotypes of rIFN-β administered mice, IFNAR-deficient mice displayed lower bacterial loads than did the WT mice at 5 days post infection (**Supplementary Figure 3B**). Our ongoing hypothesis is that, unlike the pre-exposure of rIFN-β, which is a single excessive stimulation, type I IFN signal is permanently blocked in IFNAR-deficient mice. Therefore, it could lead to a decrease in NO production in the incipient innate immune response. However, it would possible that the absence of type I IFN signal affects a certain adaptive immune response in the later phase of infection. In fact, several previous studies have reported that type I IFNs are associated with Th1-type adaptive immune response in *M. tuberculosis* infection (52–54).

Previously, our laboratory reported that NOD2 enhances the antimicrobial effect of macrophage against MAB infection by augmenting NO production (16). It has also been reported that NOD2 contributes to the induction of type I IFNs (34). In the current study, we identified the decrease in TBK1 phosphorylation and IFN-β gene expression in MAB-infected NOD2-deficient BMDMs compared with WT cells. In addition, as demonstrated in our previous study (16), NOD2-deficient mice showed higher bacterial load in lung lysates than did WT mice due to the reduced NO production. In the present study, we demonstrated that the administration of rIFN-β improves the impaired bacterial clearance of NOD2-deficient mice by increasing NO production. These results suggest the novel host defense mechanism mediated by NOD2-type I IFN–NO axis against bacterial infections.

Collectively, these data in combination with our hypothesis on IFNAR-deficient mice infection model and MAB-infected NOD2-deficient mice would also have to show lower bacterial load in lungs at 5 days post infection, because the attenuation of type I IFN signal in NOD2-deficient mice might also affect adaptive immune response, especially T-cell response (55). However, the production of type I IFNs by MAB infection is induced through not only NOD2 but also other PRRs such as toll-like receptor (TLR)2 and TLR4 (25). In fact, it has been reported that type I IFN signaling is critical for LPS-induced iNOS expression and NO production in macrophages (56) and that MAB possesses TLR-stimulating potential (25, 29, 57, 58). Therefore, it is likely that, in the early stage of infection, the attenuated type I IFN signal by the deficiency of NOD2 leads to the decrease in NO production and thus impairs the bacterial clearance. In mid-later stages of infection, however, the attenuated type I IFN signal by the NOD2 deficiency would be sufficiently compensated by the operation of other PRRs such as TLR2 and TLR4. Meanwhile, it was reported that type I IFN signaling exerts synergism in NO production through paracrine/autocrine effect of IFN-β (59). For this reason, NOD2-deficient mice would not show the same phenotypes as IFNAR-deficient mice. Currently, researches are ongoing in our laboratory to test this hypothesis.

In summary, we described here that MAB induces iNOS expression and NO production in macrophages *via* type I IFN-mediated signaling, which contribute to the intracellular growth control of the bacteria. In addition, a cytosolic receptor NOD2 is involved in MAB-induced expression of type I IFNs. The NOD2-type I IFN–NO axis may play an important role in host defense against MAB infection. However, there are several limitations in our study. Type I IFNs exert diverse biological functions on bacterial infection by modulating the expression of various cytokines as well as iNOS (5, 18). Several previous studies demonstrated that type I IFNs modulate the production of IL-10, IL-1, and TNF-α and that these cytokines influence the antimicrobial responses on *M. tuberculosis* infection by regulating the immune cell activation and recruitment (60–63). In fact, we evaluated cytokine production in the presence or absence of type I IFNs in MAB-infected BMDMs, and we confirmed that type I IFN amplified the production of TNF-α and IL-10 and inhibited the production of IL-1β (**Supplementary Figures 6A–C**). To date, the effect of TNF-α, IL-1β, and IL-10 on MAB infection is still unknown. It is necessary to further investigate the effect of these cytokines on host defense against MAB infection. In addition, although we clearly showed that the prophylactic administration of rIFN-β improves *in vivo* MAB clearance, the bacterial loads were rather lower in the lungs of IFNAR-deficient mice as compared with WT mice, suggesting that type I IFNs can exert a harmful effect on host defense against MAB infection. Further studies are strongly recommended to clarify the precise role of type I IFNs on MAB infection.

## Data Availability Statement

The datasets presented in this study can be found in online repositories. The names of the repository/repositories and accession number(s) can be found in the article/**Supplementary Material**.

## Ethics Statement

The animal study was reviewed and approved by Institutional Animal Care and the Use Committee of Chonnam National University (Approval No. CNU IACUC-YB-2017-56 and 2019-31).

## Author Contributions

J-HP and J-HA provided substantial contributions to the conception of the work. J-HA performed substantially all the experiments and data analyses and wrote the manuscript. E-JS, A-RJ, J-YP, Ye-JL, Yu-JL, and I-SS contributed to the animal experiments. D-YK, T-SL, D-HJ, and Y-JK performed the histological analysis. SS performed the bacterial culture. S-JY wrote the manuscript. All authors approved the final version of this manuscript to be published and agreed to be accountable for all aspects of the work in ensuring that questions related to the accuracy or integrity of any part of the work are appropriately investigated and resolved.

## Funding

This research was supported by the National Research Foundation of Korea (NRF) grant funded by the Korean government (MSIT) (Grant No: NRF-2018R1A2B3004143 and NRF-2017M3A9D5A0105244).

## Conflict of Interest

The authors declare that the research was conducted in the absence of any commercial or financial relationships that could be construed as a potential conflict of interest.

## Publisher’s Note

All claims expressed in this article are solely those of the authors and do not necessarily represent those of their affiliated organizations, or those of the publisher, the editors and the reviewers. Any product that may be evaluated in this article, or claim that may be made by its manufacturer, is not guaranteed or endorsed by the publisher.

## References

[1] Liao C-H Lai C-C Ding L Hou S Chiu H-C Chang S-C. Skin and Soft Tissue Infection Caused by Non-Tuberculous Mycobacteria. Int J Tuberc Lung Dis (2007) 11(1):96–102. 17217137

[2] Sanguinetti M Ardito F Fiscarelli E La Sorda M D'Argenio P Ricciotti G. Fatal Pulmonary Infection Due to Multidrug-Resistant *Mycobacterium Abscessus* in a Patient With Cystic Fibrosis. J Clin Microbiol (2001) 39(2):816–9. doi: 10.1128/JCM.39.2.816-819.2001 PMC87830 11158161

[3] Koh WJ Jeong BH Kim SY Jeon K Park KU Jhun BW. Mycobacterial Characteristics and Treatment Outcomes in *Mycobacterium Abscessus* Lung Disease. Clin Infect Dis (2017) 64(3):309–16. doi: 10.1093/cid/ciw724 28011608

[4] Kaufmann SH. Immunity to Intracellular Bacteria. Annu Rev Immunol (1993) 11(1):129–63. doi: 10.1146/annurev.iy.11.040193.001021 8476559

[5] Kalliolias GD Ivashkiv LB. Overview of the Biology of Type I Interferons. Arthritis Res Ther (2010) 12(1):S1. doi: 10.1186/ar2881 20392288 PMC2991774

[6] Honda K Takaoka A Taniguchi T. Type I Interferon [Corrected] Gene Induction by the Interferon Regulatory Factor Family of Transcription Factors. Immunity (2006) 25(3):349–60. doi: 10.1016/j.immuni.2006.08.009 16979567

[7] Platanias LC. Mechanisms of Type-I- and Type-II-Interferon-Mediated Signalling. Nat Rev Immunol (2005) 5(5):375–86. doi: 10.1038/nri1604 15864272

[8] Ting JP-Y Lovering RC Alnemri ES Bertin J Boss JM Davis BK. The NLR Gene Family: A Standard Nomenclature. Immunity (2008) 28(3):285–7. doi: 10.1016/j.immuni.2008.02.005 PMC2630772 18341998

[9] Moreira LO Zamboni DS. NOD1 and NOD2 Signaling in Infection and Inflammation. Front Immunol (2012) 3:328. doi: 10.3389/fimmu.2012.00328 23162548 PMC3492658

[10] Raymond JB Mahapatra S Crick DC Pavelka MS. Identification of the namH Gene, Encoding the Hydroxylase Responsible for the N-Glycolylation of the Mycobacterial Peptidoglycan. J Biol Chem (2005) 280(1):326–33. doi: 10.1074/jbc.M411006200 15522883

[11] Coulombe F Divangahi M Veyrier F de Léséleuc L Gleason JL Yang Y. Increased NOD2-Mediated Recognition of N-Glycolyl Muramyl Dipeptide. J Exp Med (2009) 206(8):1709–16. doi: 10.1084/jem.20081779 PMC2722178 19581406

[12] Brooks MN Rajaram MV Azad AK Amer AO Valdivia-Arenas MA Park JH. NOD2 Controls the Nature of the Inflammatory Response and Subsequent Fate of Mycobacterium Tuberculosis and M. Bovis BCG in Human Macrophages. Cell Microbiol (2011) 13(3):402–18. doi: 10.1111/j.1462-5822.2010.01544.x PMC3259431 21040358

[13] Ferwerda G Girardin SE Kullberg B-J Le Bourhis L De Jong DJ Langenberg DM. NOD2 and Toll-Like Receptors Are Nonredundant Recognition Systems of Mycobacterium Tuberculosis. PloS Pathog (2005) 1(3):e34. doi: 10.1371/journal.ppat.0010034 PMC1291354 16322770

[14] Landes MB Rajaram MV Nguyen H Schlesinger LS. Role for NOD2 in Mycobacterium Tuberculosis-Induced iNOS Expression and NO Production in Human Macrophages. J Leukoc Biol (2015) 97(6):1111–9. doi: 10.1189/jlb.3A1114-557R PMC4438743 25801769

[15] Divangahi M Mostowy S Coulombe F Kozak R Guillot L Veyrier F. NOD2-Deficient Mice Have Impaired Resistance to Mycobacterium Tuberculosis Infection Through Defective Innate and Adaptive Immunity. J Immunol (2008) 181(10):7157–65. doi: 10.4049/jimmunol.181.10.7157 18981137

[16] Lee JY Lee MS Kim DJ Yang SJ Lee SJ Noh EJ. Nucleotide-Binding Oligomerization Domain 2 Contributes to Limiting Growth of *Mycobacterium Abscessus* in the Lung of Mice by Regulating Cytokines and Nitric Oxide Production. Front Immunol (2017) 8:1477. doi: 10.3389/fimmu.2017.01477 29163541 PMC5681718

[17] Boxx GM Cheng G. The Roles of Type I Interferon in Bacterial Infection. Cell Host Microbe (2016) 19(6):760–9. doi: 10.1016/j.chom.2016.05.016 PMC5847370 27281568

[18] McNab F Mayer-Barber K Sher A Wack A O'Garra A. Type I Interferons in Infectious Disease. Nat Rev Immunol (2015) 15(2):87–103. doi: 10.1038/nri3787 25614319 PMC7162685

[19] Watanabe T Asano N Fichtner-Feigl S Gorelick PL Tsuji Y Matsumoto Y. NOD1 Contributes to Mouse Host Defense Against Helicobacter Pylori *via* Induction of Type I IFN and Activation of the ISGF3 Signaling Pathway. J Clin Invest (2010) 120(5):1645–62. doi: 10.1172/JCI39481 PMC2860924 20389019

[20] Kelly-Scumpia KM Scumpia PO Delano MJ Weinstein JS Cuenca AG Wynn JL. Type I Interferon Signaling in Hematopoietic Cells is Required for Survival in Mouse Polymicrobial Sepsis by Regulating CXCL10. J Exp Med (2010) 207(2):319–26. doi: 10.1084/jem.20091959 PMC2822595 20071504

[21] O'Connell RM Saha SK Vaidya SA Bruhn KW Miranda GA Zarnegar B. Type I Interferon Production Enhances Susceptibility to Listeria Monocytogenes Infection. J Exp Med (2004) 200(4):437–45. doi: 10.1084/jem.20040712 PMC2211937 15302901

[22] Dorhoi A Yeremeev V Nouailles G Weiner J 3rd Jorg S Heinemann E. Type I IFN Signaling Triggers Immunopathology in Tuberculosis-Susceptible Mice by Modulating Lung Phagocyte Dynamics. Eur J Immunol (2014) 44(8):2380–93. doi: 10.1002/eji.201344219 PMC4298793 24782112

[23] Ruangkiattikul N Nerlich A Abdissa K Lienenklaus S Suwandi A Janze N. cGAS-STING-TBK1-IRF3/7 Induced Interferon-Beta Contributes to the Clearing of non Tuberculous Mycobacterial Infection in Mice. Virulence (2017) 8(7):1303–15. doi: 10.1080/21505594.2017.1321191 PMC5711412 28422568

[24] Moreira-Teixeira L Mayer-Barber K Sher A O'Garra A. Type I Interferons in Tuberculosis: Foe and Occasionally Friend. J Exp Med (2018) 215(5):1273–85. doi: 10.1084/jem.20180325 PMC5940272 29666166

[25] Ruangkiattikul N Rys D Abdissa K Rohde M Semmler T Tegtmeyer PK. Type I Interferon Induced by TLR2-TLR4-MyD88-TRIF-IRF3 Controls *Mycobacterium Abscessus* Subsp. Abscessus Persistence in Murine Macrophages *via* Nitric Oxide. Int J Med Microbiol (2019) 309(5):307–18. doi: 10.1016/j.ijmm.2019.05.007 31178418

[26] Kim B-R Kim B-J Kook Y-H Kim B-J. Phagosome Escape of Rough *Mycobacterium Abscessus* Strains in Murine Macrophage *via* Phagosomal Rupture can Lead to Type I Interferon Production and Their Cell-to-Cell Spread. Front Immunol (2019) 10:125. doi: 10.3389/fimmu.2019.00125 30766538 PMC6365470

[27] Celada A Gray PW Rinderknecht E Schreiber R. Evidence for a Gamma-Interferon Receptor That Regulates Macrophage Tumoricidal Activity. J Exp Med (1984) 160(1):55–74. doi: 10.1084/jem.160.1.55 6330272 PMC2187421

[28] Nayak DK Mendez O Bowen S Mohanakumar T. Isolation and *In Vitro* Culture of Murine and Human Alveolar Macrophages. J Vis Exp: JoVE (2018) 134). doi: 10.3791/57287 PMC6100701 29733312

[29] Kim J-S Kang M-J Kim WS Han SJ Kim HM Kim HW. Essential Engagement of Toll-Like Receptor 2 in Initiation of Early Protective Th1 Response Against Rough Variants of *Mycobacterium Abscessus* . Infect Immun (2015) 83(4):1556–67. doi: 10.1128/IAI.02853-14 PMC4363419 25644006

[30] Green LC Wagner DA Glogowski J Skipper PL Wishnok JS Tannenbaum SR. Analysis of Nitrate, Nitrite, and [15N] Nitrate in Biological Fluids. Anal Biochem (1982) 126(1):131–8. doi: 10.1016/0003-2697(82)90118-X 7181105

[31] Choi J-H Jo S-G Jung S-K Park W-T Kim K-Y Park Y-W. Immunomodulatory Effects of Ethanol Extract of Germinated Ice Plant (Mesembryanthemum Crystallinum). Lab Anim Res (2017) 33(1):32–9. doi: 10.5625/lar.2017.33.1.32 PMC5385280 28400837

[32] Weiss G Schaible UE. Macrophage Defense Mechanisms Against Intracellular Bacteria. Immunol Rev (2015) 264(1):182–203. doi: 10.1111/imr.12266 25703560 PMC4368383

[33] Chau T da Silva J Ghaffari A Zelazny A Olivier K. Synergistic Effect of Nitric Oxide With Antibiotics Against *Mycobacterium Abscessus In Vitro* . B19 Adv IN THE Treat OF NTM: Am Thorac Society; (2019) p:A2656–A. doi: 10.1164/ajrccm-conference.2019.199.1_MeetingAbstracts.A2656

[34] Pandey AK Yang Y Jiang Z Fortune SM Coulombe F Behr MA. NOD2, RIP2 and IRF5 Play a Critical Role in the Type I Interferon Response to Mycobacterium Tuberculosis. PloS Pathog (2009) 5(7):e1000500. doi: 10.1371/journal.ppat.1000500 19578435 PMC2698121

[35] Banks DA Ahlbrand SE Hughitt VK Shah S Mayer-Barber KD Vogel SN. Mycobacterium Tuberculosis Inhibits Autocrine Type I IFN Signaling to Increase Intracellular Survival. J Immunol (2019) 202(8):2348–59. doi: 10.4049/jimmunol.1801303 PMC6456408 30833347

[36] Miller BH Fratti RA Poschet JF Timmins GS Master SS Burgos M. Mycobacteria Inhibit Nitric Oxide Synthase Recruitment to Phagosomes During Macrophage Infection. Infect Immun (2004) 72(5):2872–8. doi: 10.1128/IAI.72.5.2872-2878.2004 PMC387846 15102799

[37] Bonay M Roux A Floquet J Retory Y Herrmann J Lofaso F. Caspase-Independent Apoptosis in Infected Macrophages Triggered by Sulforaphane *via* Nrf2/p38 Signaling Pathways. Cell Death Discov (2015) 1(1):1–10. doi: 10.1038/cddiscovery.2015.22 PMC4979433 27551455

[38] Kim B-R Kim B-J Kook Y-H Kim B-J. *Mycobacterium Abscessus* Infection Leads to Enhanced Production of Type 1 Interferon and NLRP3 Inflammasome Activation in Murine Macrophages *via* Mitochondrial Oxidative Stress. PloS Pathog (2020) 16(3):e1008294. doi: 10.1371/journal.ppat.1008294 32210476 PMC7094820

[39] Whang J Back YW Lee K-I Fujiwara N Paik S Choi CH. *Mycobacterium Abscessus* Glycopeptidolipids Inhibit Macrophage Apoptosis and Bacterial Spreading by Targeting Mitochondrial Cyclophilin D. Cell Death Dis (2017) 8(8):e3012–e. doi: 10.1038/cddis.2017.420 PMC5596598 28837151

[40] Cobbold S Waldmann H. Infectious Tolerance. Curr Opin Immunol (1998) 10(5):518–24. doi: 10.1016/S0952-7915(98)80217-3 9794831

[41] Couper KN Blount DG Riley EM. IL-10: The Master Regulator of Immunity to Infection. J Immunol (2008) 180(9):5771–7. doi: 10.4049/jimmunol.180.9.5771 18424693

[42] Kotas ME Medzhitov R. Homeostasis, Inflammation, and Disease Susceptibility. Cell (2015) 160(5):816–27. doi: 10.1016/j.cell.2015.02.010 PMC4369762 25723161

[43] Wallach D Kang T-B Kovalenko A. Concepts of Tissue Injury and Cell Death in Inflammation: A Historical Perspective. Nat Rev Immunol (2014) 14(1):51–9. doi: 10.1038/nri3561 24336099

[44] Bogdanovski K Chau T Robinson CJ MacDonald SD Peterson AM Mashek CM. Antibacterial Activity of High-Dose Nitric Oxide Against Pulmonary *Mycobacterium Abscessus* Disease. Access Microbiol (2020) 2(9):acmi000154. doi: 10.1099/acmi.0.000154 33195983 PMC7656188

[45] Bentur L Gur M Ashkenazi M Livnat-Levanon G Mizrahi M Tal A. Pilot Study to Test Inhaled Nitric Oxide in Cystic Fibrosis Patients With Refractory *Mycobacterium Abscessus* Lung Infection. J Cystic Fibrosis (2020) 19(2):225–31. doi: 10.1016/j.jcf.2019.05.002 31129068

[46] Yaacoby-Bianu K Gur M Toukan Y Nir V Hakim F Geffen Y. Compassionate Nitric Oxide Adjuvant Treatment of Persistent Mycobacterium Infection in Cystic Fibrosis Patients. Pediatr Infect Dis J (2018) 37(4):336–8. doi: 10.1097/INF.0000000000001780 28885458

[47] Bentur L Masarweh K Livnat-Levanon G Ashkenazi M Dagan A Mizrahi M. Nitric Oxide Inhalations in CF Patients Infected With *Mycobacterium Abscessus* Complex: A Prospective, Open-Labeled, Multi-Center Pilot Study. C96 Adv IN THE Manage OF Pulmonary NTM Dis: Am Thorac Society; (2018) p:A5919–A.

[48] Schyns J Bureau F Marichal T. Lung Interstitial Macrophages: Past, Present, and Future. J Immunol Res (2018) 2018:5160794. doi: 10.1155/2018/5160794 29854841 PMC5952507

[49] Sabatel C Radermecker C Fievez L Paulissen G Chakarov S Fernandes C. Exposure to Bacterial CpG DNA Protects From Airway Allergic Inflammation by Expanding Regulatory Lung Interstitial Macrophages. Immunity (2017) 46(3):457–73. doi: 10.1016/j.immuni.2017.02.016 28329706

[50] Bedoret D Wallemacq H Marichal T Desmet C Calvo FQ Henry E. Lung Interstitial Macrophages Alter Dendritic Cell Functions to Prevent Airway Allergy in Mice. J Clin Invest (2009) 119(12):3723–38. doi: 10.1172/JCI39717 PMC2786798 19907079

[51] Franke-Ullmann G Pförtner C Walter P Steinmüller C Lohmann-Matthes M-L Kobzik L. Characterization of Murine Lung Interstitial Macrophages in Comparison With Alveolar Macrophages *In Vitro* . J Immunol (1996) 157(7):3097–104. 8816420

[52] Manca C Tsenova L Bergtold A Freeman S Tovey M Musser JM. Virulence of a Mycobacterium Tuberculosis Clinical Isolate in Mice is Determined by Failure to Induce Th1 Type Immunity and is Associated With Induction of IFN-α/β. Proc Natl Acad Sci (2001) 98(10):5752–7. doi: 10.1073/pnas.091096998 PMC33285 11320211

[53] Ordway D Henao-Tamayo M Harton M Palanisamy G Troudt J Shanley C. The Hypervirulent Mycobacterium Tuberculosis Strain HN878 Induces a Potent TH1 Response Followed by Rapid Down-Regulation. J Immunol (2007) 179(1):522–31. doi: 10.4049/jimmunol.179.1.522 17579073

[54] Manca C Tsenova L Freeman S Barczak AK Tovey M Murray PJ. Hypervirulent M. Tuberculosis W/Beijing Strains Upregulate Type I IFNs and Increase Expression of Negative Regulators of the Jak-Stat Pathway. J Interferon Cytokine Res (2005) 25(11):694–701. doi: 10.1089/jir.2005.25.694 16318583

[55] Mourik BC Lubberts E de Steenwinkel JE Ottenhoff TH Leenen PJ. Interactions Between Type 1 Interferons and the Th17 Response in Tuberculosis: Lessons Learned From Autoimmune Diseases. Front Immunol (2017) 8:294. doi: 10.3389/fimmu.2017.00294 28424682 PMC5380685

[56] Vadiveloo PK Vairo G Hertzog P Kola I Hamilton JA. Role of Type I Interferons During Macrophage Activation by Lipopolysaccharide. Cytokine (2000) 12(11):1639–46. doi: 10.1006/cyto.2000.0766 11052814

[57] Sampaio EP Elloumi HZ Zelazny A Ding L Paulson ML Sher A. *Mycobacterium Abscessus* and M. Avium Trigger Toll-Like Receptor 2 and Distinct Cytokine Response in Human Cells. Am J Respir Cell Mol Biol (2008) 39(4):431–9. doi: 10.1165/rcmb.2007-0413OC PMC2551704 18441280

[58] Shin DM Yang CS Yuk JM Lee JY Kim KH Shin SJ. *Mycobacterium Abscessus* Activates the Macrophage Innate Immune Response *via* a Physical and Functional Interaction Between TLR2 and Dectin-1. Cell Microbiol (2008) 10(8):1608–21. doi: 10.1111/j.1462-5822.2008.01151.x 18373632

[59] Whitmore MM DeVeer MJ Edling A Oates RK Simons B Lindner D. Synergistic Activation of Innate Immunity by Double-Stranded RNA and CpG DNA Promotes Enhanced Antitumor Activity. Cancer Res (2004) 64(16):5850–60. doi: 10.1158/0008-5472.CAN-04-0063 15313929

[60] Novikov A Cardone M Thompson R Shenderov K Kirschman KD Mayer-Barber KD. Mycobacterium Tuberculosis Triggers Host Type I IFN Signaling to Regulate IL-1β Production in Human Macrophages. J Immunol (2011) 187(5):2540–7. doi: 10.4049/jimmunol.1100926 PMC3159798 21784976

[61] McNab FW Ewbank J Howes A Moreira-Teixeira L Martirosyan A Ghilardi N. Type I IFN Induces IL-10 Production in an IL-27–Independent Manner and Blocks Responsiveness to IFN-γ for Production of IL-12 and Bacterial Killing in Mycobacterium Tuberculosis–Infected Macrophages. J Immunol (2014) 193(7):3600–12. doi: 10.4049/jimmunol.1401088 PMC4170673 25187652

[62] Mayer-Barber KD Andrade BB Barber DL Hieny S Feng CG Caspar P. Innate and Adaptive Interferons \ress IL-1α and IL-1β Production by Distinct Pulmonary Myeloid Subsets During Mycobacterium Tuberculosis Infection. Immunity (2011) 35(6):1023–34. doi: 10.1016/j.immuni.2011.12.002 PMC3246221 22195750

[63] Bernut A Nguyen-Chi M Halloum I Herrmann J-L Lutfalla G Kremer L. *Mycobacterium Abscessus*-Induced Granuloma Formation Is Strictly Dependent on TNF Signaling and Neutrophil Trafficking. PloS Pathog (2016) 12(11):e1005986. doi: 10.1371/journal.ppat.1005986 27806130 PMC5091842

