# Supplementary material for: Type I Interferons Are Involved in the Intracellular Growth Control of Mycobacterium abscessus by Mediating NOD2-Induced Production of Nitric Oxide in Macrophages
Source: Front Immunol. 2021 Oct 28;12:738070. doi: 10.3389/fimmu.2021.738070 (PMC8581665; doi:10.3389/fimmu.2021.738070)
Supplement: Supplementary file 1 [file DataSheet_1.docx]

Supplementary Material

##
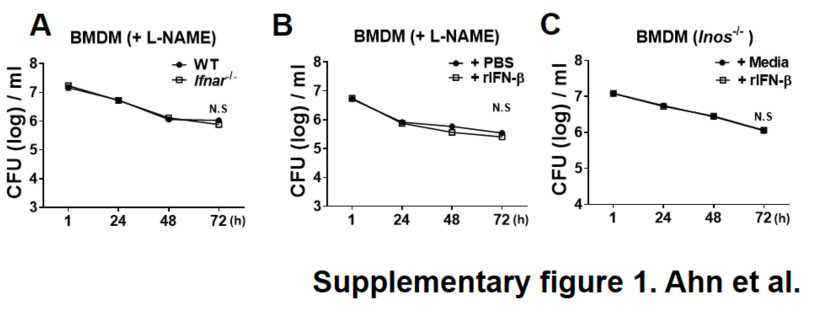


**Supplementary figure 1.** Type 1 IFNs did not inhibits the intracellular MAB growth in the absence of NO in macrophages

**(A)** BMDMs were infected with MAB at MOI 1:25 present of L-NAME (iNOS inhibitor, 1 mM, 2 h pre-treated). **(B)** BMDMs were infected with present of L-NAME and absent or present of rIFN-β (1000 unit/ml, 2 h pre-treated). **(C)** *Inos*^-/-^ BMDMs were infected with MAB absent or present of rIFN-β. Intracellular bacterial CFU on indicated times were evaluated by intracellular bacterial growth assay. **(A-C)** The results are from one representative experiment of two independent experiments (NS: Not Statistically Significant).


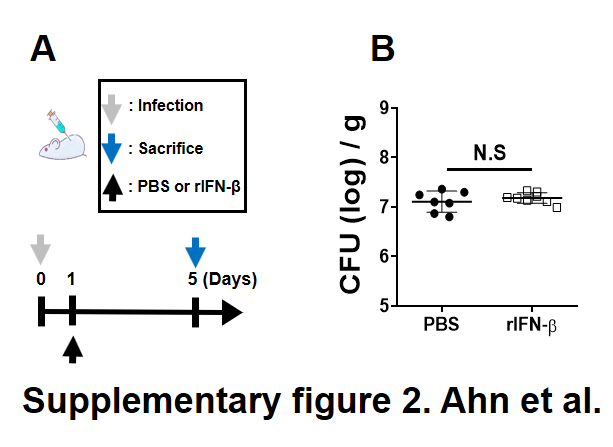


**Supplementary figure 2.** The effect of post-infection administration of rIFN-β on bacterial clearance in MAB-infected lungs of mice.

**(A)** The administration of rIFN-β and bacterial infection were performed according to the schedule indicated in diagram. **(B)**. Mice were infected with 2×10^7^ CFU of MAB per mouse intranasally. After 1day mice were administrated with PBS or rIFN-β (800 units per mouse) intranasally under anesthesia. The bacterial loads in the lung lysate were determined at 5 days post infection. **(B)** The results are one representative data of two independent experiments (n=7-8), (NS: Not Statistically Significant).


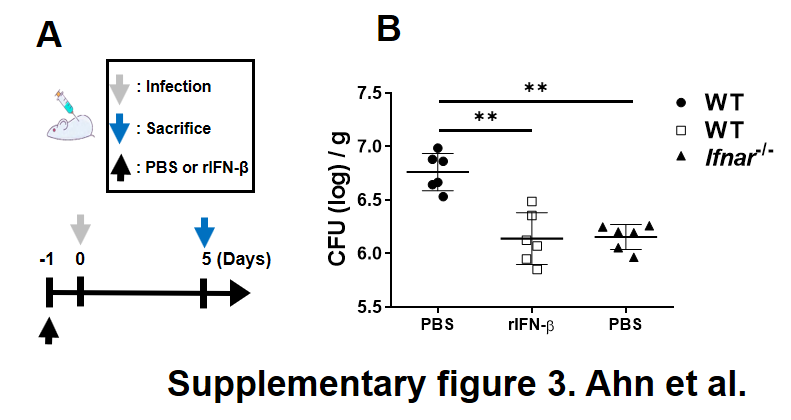


**Supplementary figure 3.** The comparison of bacterial clearance in MAB-infected lungs of WT and *Ifnar*^-/-^ mice.

**(A)** The administration of rIFN-β and bacterial infection were performed according to the schedule indicated in diagram. **(B)** Mice were administrated with PBS or rIFN-β (800 units per mouse) intranasally under anesthesia. After 1day, mice were infected with 2×10^7^ CFU of MAB per mouse intranasally. The bacterial loads in the lung lysate were determined at 5 days post infection. **(B)** The results are one representative data of two independent experiments (n=6). (**P<0.01).


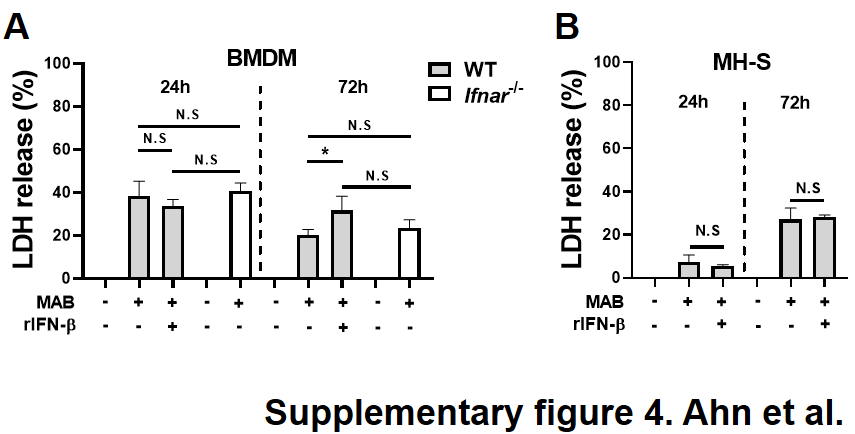


**Supplementary figure 4.** Comparison of MAB-induced LDH releases according to the presence or absence of type 1 IFN signaling.

**(A, B)** BMDMs and MH-S cells were infected with MAB at MOI 1:25 absent or present of rIFN-β (1000 unit/ml, 2 h pre-treated). At indicated times, the levels of lactate dehydrogenase (LDH) in cell culture supernatant were determined by colorimetric alternative to radioactive cytotoxicity assay. **(A, B)** The results are from one representative experiment of two independent experiments (*P<0.05, NS: Not Statistically Significant).


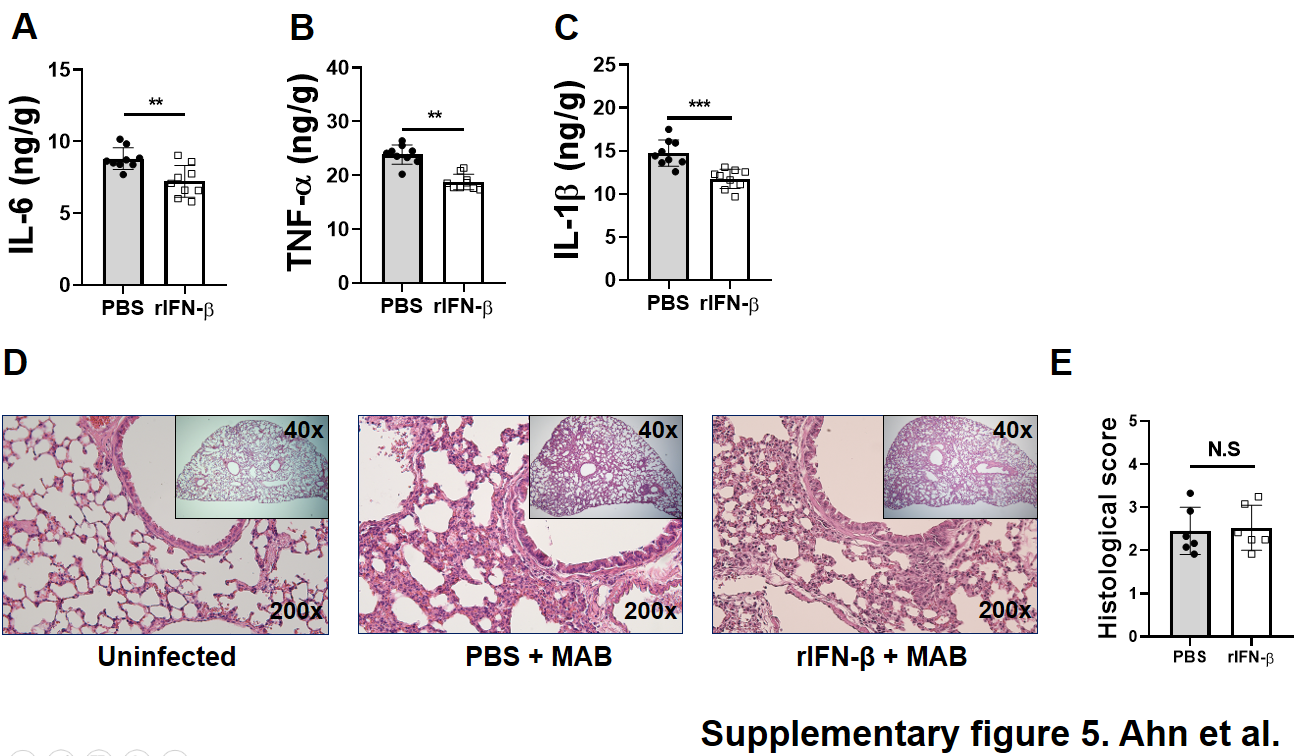


**Supplementary figure 5.** Effect of type 1 IFN on inflammatory changes in MAB-infected lungs of mice.

**(A-C)**. IL-6, TNF-α and IL-1β levels at 5 days post infection in the lung lysate were determined by ELISA. **(D, E)** H&E stained lung sections on 5 days post infection are shown as microscopic images **(**magnification 40 and 200). Histological score of lung inflammatory lesion were determined as a value 0 to 5 points. **(A-C)** The results are merged data of two independent experiments (n=4-5). **(D, E)** The results are one representative data of two independent experiments (n=6) (**P<0.01, ***P<0.001, NS: Not Statistically Significant).


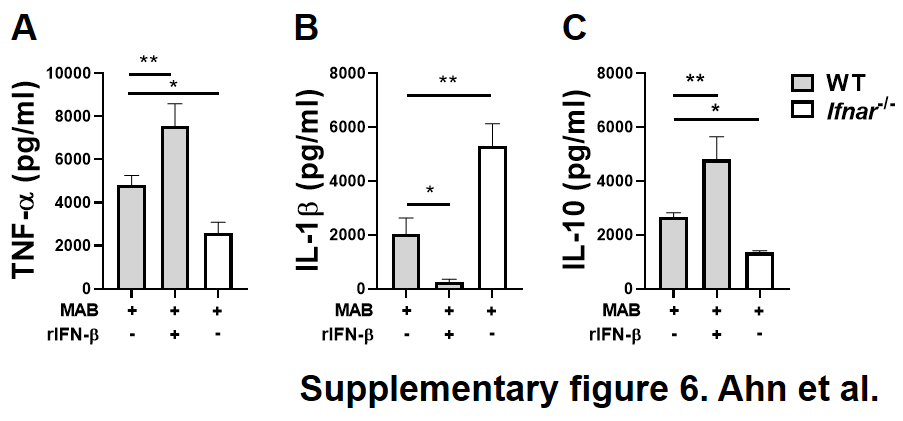


**Supplementary figure 6.** Comparison of MAB-induced cytokine production according to the presence or absence of type 1 IFN signaling in BMDMs.

**(A-C)** WT and *Ifnar*^-/-^ BMDMs were infected with MAB at MOI 1:25 absent or present of rIFN-β (1000 unit/ml, 2 h pre-treated). After 24 h infection, the levels of indicated cytokine in cell culture supernatant were determined by ELISA. **(A-C)** The results are from one representative experiment of two independent experiments (*P<0.05, **P<0.01).
